# The Relationship Between Academic Stress, Sleep Quality, and Psychological Wellbeing in Pasifika and New Zealand European Students at the University of Otago

**DOI:** 10.1007/s40615-024-02043-8

**Published:** 2024-06-10

**Authors:** Willie Solomona Time, Ilaisaane Foli Fakapulia, Latika Samalia, Erik Wibowo

**Affiliations:** 1 Department of Anatomy, University of Otago, Dunedin, 9016 New Zealand; 2 School of Medical Sciences, University of Sydney, Sydney, 2006 Australia

**Keywords:** Pasifika students, New Zealand European students, Sleep quality, Psychological wellbeing, Academic stress, Ethnic differences

## Abstract

**Background:**

Pasifika students in New Zealand are overrepresented in poor academic outcomes, and their academic challenges may potentially influence their wellbeing. We aim to: 1) compare the academic stress, sleep quality, and psychological wellbeing of Pasifika and New Zealand European (NZE) anatomy students at the University of Otago, and 2) determine if academic stress mediates the association between their psychological wellbeing and sleep quality.

**Methods:**

We launched a brief online survey to Pasifika and NZE students in our department. The survey included basic demographics, Perception of Academic Stress Scale, Pittsburgh Sleep Quality Index, Hospital Anxiety and Depression Scale, a short loneliness scale, a reduced morningness-eveningness scale, and the Epworth Sleepiness Scale.

**Results:**

Perceived academic stress were comparable between NZE and Pasifika students, but Pasifika students reported poorer sleep quality than NZE students (t(113) = 14.41, *P* < .001). In addition, Pasifika students reported more loneliness (t(119) = 8.933, *P* < .001), less anxiety symptoms (t(120) = 2.469, *P* = .015), and less of a morning person (t(121) = 2.618, *P* = .010) than NZE students, but they had comparable depressive symptoms and daytime sleepiness. After controlling for age, ethnicity and gender, we found that academic stress fully mediated the relationship between anxiety or depressive symptoms and poor sleep quality. Furthermore, academic stress partially mediated the relationship between loneliness and poor sleep quality in our cohort.

**Conclusion:**

Our findings highlight the importance for academic institutions to support students’ wellbeing, including ethnic minority students such as Pasifika students in New Zealand.

## Introduction

University students may experience stress due to academic and non-academic reasons [1]. Students have academic stressors such as from catching up with lectures, preparing for exams and doing assignments. Students may also have stressors due to sociocultural (e.g., adapting to a new place, forming new friendships) or health (e.g., medical, psychological) reasons. These stressors may potentially affect their sleep and psychological wellbeing while they study. As an example, medical students are prone to having insomnia symptoms during their studies [2–4]. These insomnia symptoms are also associated with increased risk of depressive symptoms [2]. However students may not always be able to manage their sleep due to their heavy workload and busy schedule [5].

Ethnic minority university students may be at higher risks of having poor sleep. Black students in the United States, for example, have higher rate of having short sleep duration than White students but their rates of insomnia symptoms are comparable [6]. Hispanic students also have poorer sleep quality than White students [7]. Another study further shows that there are higher proportions of White university students who get sufficient sleep than Asian, Black, and Hispanic students [8].

Various factors may influence sleep in university students. Biological sex has been known to influence sleep; e.g., insomnia prevalence is higher in females than in males starting from puberty onwards [9]. Health conditions can also affect sleep in university students. As an example, obesity is linked to short sleep duration [10]. Socio-cultural factors may potentially affect sleep too. For example, students who work part-time may have reduced sleep duration and experience excessive daytime sleepiness [11]. Overall these findings highlight the importance of supporting student wellbeing during their studies, especially those with elevated risks of having sleep issues.

### Pasifika Students in New Zealand

In New Zealand, about 8% of the population are Pacific peoples based on the 2018 census [12]. The term “Pasifika” in New Zealand refers to indigenous people of the Pacific Islands. They either had migrated from the Pacific Islands or descendants of people who migrated from the Pacific region to New Zealand [13]. There are similarities and differences among the many Pacific ethnic groups in their cultures, languages and experience.

In New Zealand universities, unlike New Zealand European (NZE) students, Pasifika students are present as ethnic minority students. As an example, internal data from the University of Otago show that about 9% of the medical students are Pasifika. In addition, there are approximately 90-100 Pasifika students who take undergraduate anatomy courses in the Department of Anatomy [14]. In our recent study [15], there are three main reasons for Pasifika students to study at the University of Otago. First, they prefer the education programs and scholarships that are offered by the university. Second, other people (e.g., family members, siblings) recommend the University of Otago as a place for their tertiary education. Third, some Pasifika students personally prefer to study away from home. Currently, however, there is no published information on the difference in their academic stress, sleep quality, and psychological wellbeing as compared to those of NZE students.

In our recent 5-year retrospective review on students’ academic performance [14], Pasifika students are more likely to achieve lower marks in anatomy courses than NZE students. This finding is consistent with data from other studies in New Zealand which indicate ethnic disparity in academic performance between Pasifika and NZE healthcare professional [16], undergraduate science [17], and first-year undergraduate anatomy [18] students. Thus, there is a possibility that Pasifika students in New Zealand may have elevated academic stress, and this stress potentially influence their sleep and psychological wellbeing.

Recently, we conducted several studies related to the experience of Pasifika students at the University of Otago. Majority of Pasifika students in the Department of Anatomy at the University of Otago come from other parts of New Zealand that have higher proportion of Pacific peoples [14, 19] so some experience a culture shock when they move to Dunedin because of the small proportion of Pacific peoples in the community [15]. Family relationship is important in Pacific cultures so, for many, being away from family and home also means being distant from their grounded culture, and this shift in living arrangement may influence their daily living in Dunedin. Considering that religion is an important aspect in Pacific cultures [20], some Pasifika students establish new connections with Pacific peoples in Dunedin through church activities.

In term of their education experience, male Pasifika students perceive higher stress levels related to academic expectation than female students [19]. Furthermore, social anxiety as ethnic minority, higher religiosity and lower perception of social provision correlate with higher academic stress among Pasifika students in the Department of Anatomy at the University of Otago [19]. In another study, we also show that >70% of Pasifika students in the Department of Anatomy at the University of Otago experience insomnia symptoms (39% were moderate to severe), and that their academic stress is associated with more severe insomnia symptoms [21].

Currently, there are limited data on the sleep quality of Pasifika students in higher education, but there is a possibility that Pasifika students may have poorer sleep than NZE students as findings from other populations show such a trend. For example, data from the general population show that Pacific people have a higher rate of short-sleepers (< 7 hr a night) than NZE people [22]. Among high school students in New Zealand, the rate of students with sleep problems for more than one month do not differ between ethnicities [23] but their analysis was based on a single Yes/No question which is not specific (i.e., “Do you have problems getting to sleep, staying asleep or waking early such that it affects your work function the next day—this includes feeling excessively sleepy the next day?”). In another study [24], only 26.6% of Pasifika adolescents sleep at least 8 hours over the past week. Pasifika adolescents also have later bedtime [25] and shorter sleep during the weekends [26] than NZE adolescents.

Psychological wellbeing may potentially influence sleep among Pasifika students as previous studies have highlighted the complex relationship between psychological wellbeing and sleep among university students. As an example, depressive and anxiety symptoms are related to insomnia symptoms among university students [2, 27–29]. Loneliness has also been found to correlate with poor sleep among university students [30, 31]. In addition, evening chronotype is associated with elevated risks of insomnia symptoms among university students [32]. While these psychological factors can affect sleep quality, currently it is unknown whether academic stress mediates the association between psychological wellbeing and sleep quality among university students.

Using a cross-sectional design, we aim to answer the following research questions: 1) “Do academic stress, sleep quality and psychological wellbeing of Pasifika differ from those of NZE students?”, and 2) “Does academic stress mediate the relationship between psychological wellbeing and sleep quality among university students?”. Data from this study can be used as evidence to highlight the importance to support student wellbeing in higher education.

## Methods

### Recruitment

The protocol for this research was approved by the University of Otago Human Ethics Committee (D22/167). We launched a short online survey between September 2022 to April 2023. Using a convenience sampling strategy, the survey was sent via email to 93 Pasifika students and 220 NZE students who took anatomy courses at second- and third-year undergraduate levels in the Department of Anatomy at the University of Otago.

To avoid confounding ethnic variable, the NZE students in this cohort were students who only identified as “New Zealand European/Pākehā” in the University of Otago’s database. They were New Zealanders of a European background. Students who identified as “Australian”, “British/Irish”, “Dutch”, “German”, or “Other European” were not recruited. To recruit Pasifika students, we sent the survey to those students who identified at least one of the following Pacific ethnicities: “Cook Island Māori”, “Fijian”, “Samoan”, “Niuean”, “Tongan”, “Tokelauan”, or “Other Pacific Peoples”. Pasifika students who also identified as “New Zealand European/Pākehā” or other ethnicities were categorized as Pasifika. Due to the much smaller proportion of Pasifika students at the University of Otago, Pasifika students with more than one ethnicities were also recruited.

In the invitation email, we included a brief description of the study and the survey link. The landing page of the survey had the Participant Information Sheet, and Consent Form. Participants could only see the survey after they consented to the study. We built the survey on the Research Electronic Data Capture database. Participants needed around 15 minutes to fill in the survey. 

### Questionnaires

The survey included questions on:

### Demographics

Participants completed questions on age, ethnicity, gender, place of birth, place of growing up, relationship status, living condition, first language, financial distress, and whether they completed the university’s foundation program. The University of Otago has a foundation program that is aimed to prepare students with academic skills for their future university studies. While not mandatory, students who take the program may have gained additional skills that they had not learned in high schools.

### Perceived Academic Stress

Academic stress was measured using the Perception of Academic Stress Scale [1]. This questionnaire has 18 items related to academic stress, and they can be divided into three subscales for stresses related to academic expectations, academic work and examination, and students’ academic self-perception. Each item can be rated ranging from “strongly disagree” (1) to “strongly agree” (5). Five items were reverse-scored. The internal consistency in our sample was α = .856.

### Sleep Quality

Sleep quality was measured using the Pittsburgh Sleep Quality Index (PSQI) [33]. There are 19 items in this questionnaire and they can be scored to have seven components on subjective sleep quality, sleep latency, sleep duration, habitual sleep efficiency, sleep disturbances, use of sleep medication and daytime functioning. The scores of these seven components can be added to obtain a global score. A higher score indicates poorer sleep quality. The internal consistency in our sample was α = .773.

### Loneliness

Loneliness was assessed using a three-item scale [34]. Each asks about the frequency of feeling lacking companionship, feeling left out, or feeling isolated. The answer options for each item were “hardly ever” (1), “some of the time” (2), or “often” (3). A higher score indicates more loneliness. The internal consistency in our sample was α = .847.

### Anxiety and Depression

Anxiety and depressive symptoms were assessed using the Hospital Anxiety and Depression Scale [35]. The scale contains 14 items related to anxiety and depression (seven items for each subscale). Each can be rated on a four-point scale, ranging from 0 to 3. Higher scores indicate a higher level of anxiety or depressive symptoms. The internal consistencies in our sample for the anxiety and depression subscales were α = .833 and α = .831 respectively.

### Chronotype

Chronotype was measured using an abbreviated morningness-eveningness scale [36]. This scale consists of five questions about preference of time to get up, how tired they were during the first half-hour after having woken in the morning, what time in the evening they felt tired, what time during the day they felt their best, and whether they considered themselves a “morning” or “evening” person. A higher score characterises a morning person. The internal consistency in our sample was α = .694.

### Sleepiness

Daytime sleepiness was measured using the Epworth Sleepiness scale (ESS) [37]. This questionnaire asks about the likelihood of falling asleep in eight different daytime situations (e.g., when reading, talking to someone, or sitting after lunch). Each item can be rated on a scale from 0 (would never doze) to 3 (high chance of dozing), with a higher score indicating a higher chance of dozing. The internal consistency in our sample was α = .791.

### Data Analyses

At the end of the recruitment, 83 NZE students and 75 Pasifika students accessed the survey, but data from 21 and 11 students respectively were excluded due to extensive missing data. Data from 62 NZE and 64 Pasifika students were analyzed using the SPSS software (IBM, version 29). Demographic, academic stress and wellbeing data were compared based on ethnicity (NZE or Pasifika), using t-test for continuous variables, and χ^2^ test for categorical variables. Pearson correlation was performed to determine the relationship between sleep quality, psychological wellbeing and academic stress. Mediation analyses were conducted to determine whether academic stress mediates the association between psychological wellbeing and sleep quality, using the method outlined in Baron and Kenny [38], followed by the Sobel test (done online at http://quantpsy.org/sobel/sobel.htm). All mediation analyses were controlled for age, gender and ethnicity. *P* < .05 was considered significant.

## Results

Table 1 compares the demographic of our participants based on their ethnicities. Pasifika participants were on average significantly older than NZE participants (t(92) = 3.264, *P* < .001). The top three ethnicities of the Pasifika participants were Samoan (33.3%), Fijian (25.4%) and Tongan (19%). Proportionally, there were significantly more female NZE participants than Pasifika participants (χ^2^(1) = 8.343, *P *< .01), and more Pasifika participants who had done third-year courses than NZE participants (χ^2^(1) = 4.486, *P *< .05). There were significant differences in the birth location (χ^2^(3) = 20.778, *P *< .001) and place of growing up (χ^2^(3) = 11.040, *P *< .05). This is because some Pasifika participants were born (28.6%) and grew up primarily (12.7%) in Pacific Islands. Similarly fewer Pasifika participants (69.8%) had English as their first language than NZE participants (98.4%, χ^2^(1) = 18.946, *P *< .001). In addition, more Pasifika students (27.0%) had attended the University’s Foundation Program than NZE students (1.6%, χ^2^(1) = 16.318, *P *< .001), before they started their first-year university studies. Furthermore, Pasifika students reported more financial distress than NZE students (t(123) = 2.015, *P* = .023).

**Table 1:** Demographic data of participants. Data are presented as mean (standard deviation) for continuous variables, and number (percentage) for categorical variables

	NZE	Pasifika		*P* value
Age	20.4 (1.1)	21.4 (1.9)	t(92) = 3.264	.002
Ethnicities				
NZE	62 (100)	17 (27)		
Māori		5 (7.9)		
Samoan		21 (33.3)		
Fijian		16 (25.4)		
Tongan		12 (19.0)		
Cook Islands Māori		10 (15.9)		
Niuean		5 (7.9)		
Tokelauan		2 (3.2)		
Pacific People, not further defined		3 (4.8)		
Asian		8 (12.7)		
Middle Eastern/Latin American/African		2 (3.2)		
Other ethnicities		6 (9.5)		
Gender				
Male	8 (13.1)	22 (35.5)	χ^2^(1) = 8.343	.004
Female	53 (86.9)	40 (64.5)		
Had completed				
Second-year undergraduate courses	62 (100)	60 (95.2)	χ^2^(1) = 3.025	.082
Third-year undergraduate courses	19 (30.6)	31 (49.2)	χ^2^(1) = 4.486	.034
Birth location			χ^2^(3) = 20.778	< .001
Dunedin	3 (4.8)	2 (3.2)		
NZ, other than Dunedin	54 (87.1)	40 (63.5)		
Pacific Islands	0	18 (28.6)		
Outside of NZ	5 (8.1)	3 (4.8)		
Primary place of growing up			χ^2^(3) = 11.040	.012
Dunedin	5 (8.1)	2 (3.2)		
NZ, other than Dunedin	55 (88.7)	48 (76.2)		
Pacific Islands	0	8 (12.7)		
Outside of NZ	2 (3.2)	5 (7.9)		
In a relationship	23 (37.7)	23 (36.5)		
English as first language	61 (98.4)	44 (69.8)	χ^2^(1) = 18.946	< .001
Joined the University Foundation programme	1 (1.6)	17 (27.0)	χ^2^(1) = 16.318	< .001
Living condition			χ^2^(2) = 3.792	.150
In a university college	4 (6.5)	1 (1.6)		
Living alone	2 (3.2)	6 (9.5)		
Living with multiple people	56 (90.3)	56 (88.9)		
Level of financial distress (out of 10)	5.9 (2.3)	6.8 (2.6)	t(123) = 2.015	.046

### Academic Stress and Psychological Wellbeing

**Fig. 1:**
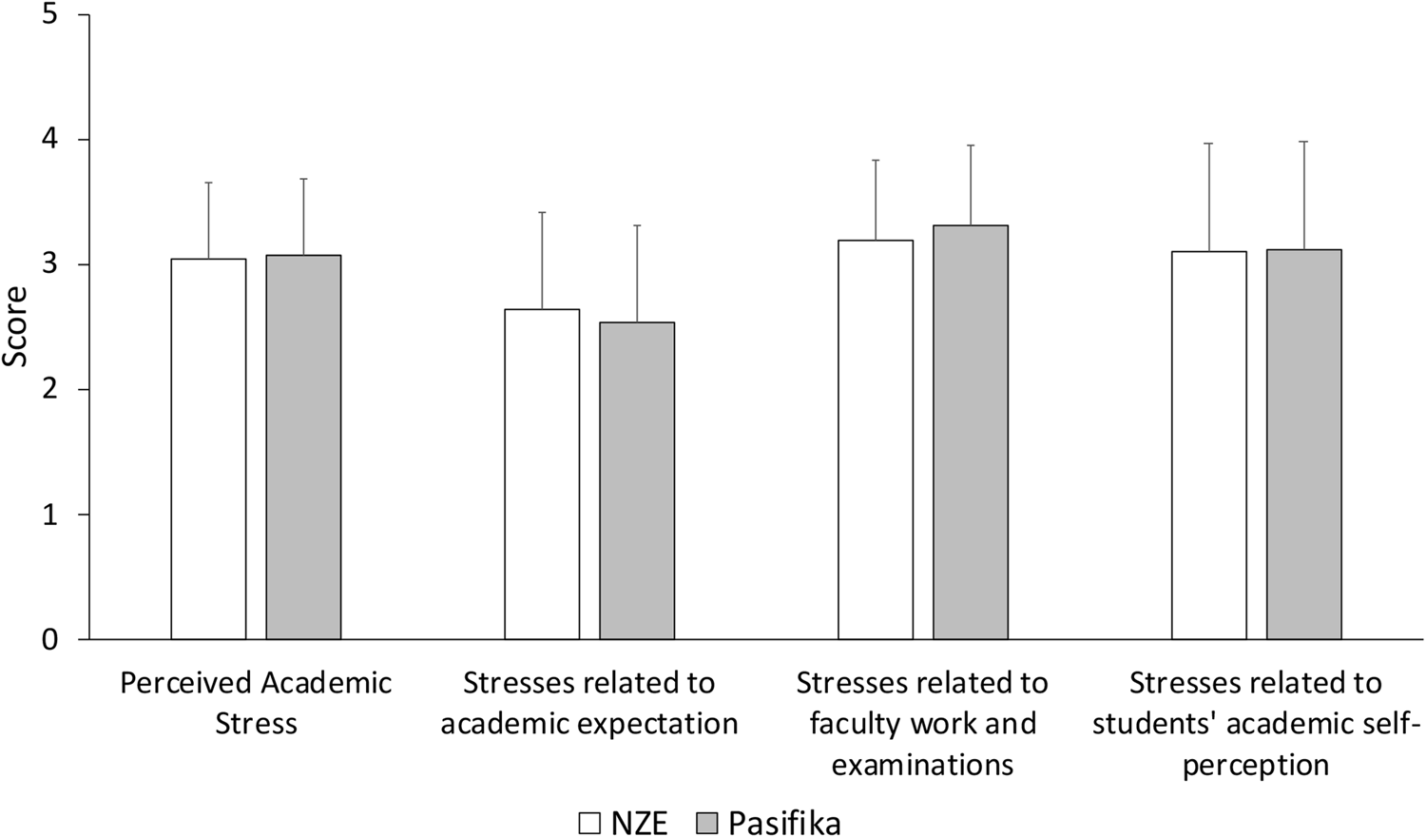
The comparison of perceived academic stress and its subscales between NZE (white bars) and Pasifika (grey bars) students. NZE and Pasifika students reported similar level of academic stresses during their studies. Error bars indicate standard deviations

**Fig. 2:**
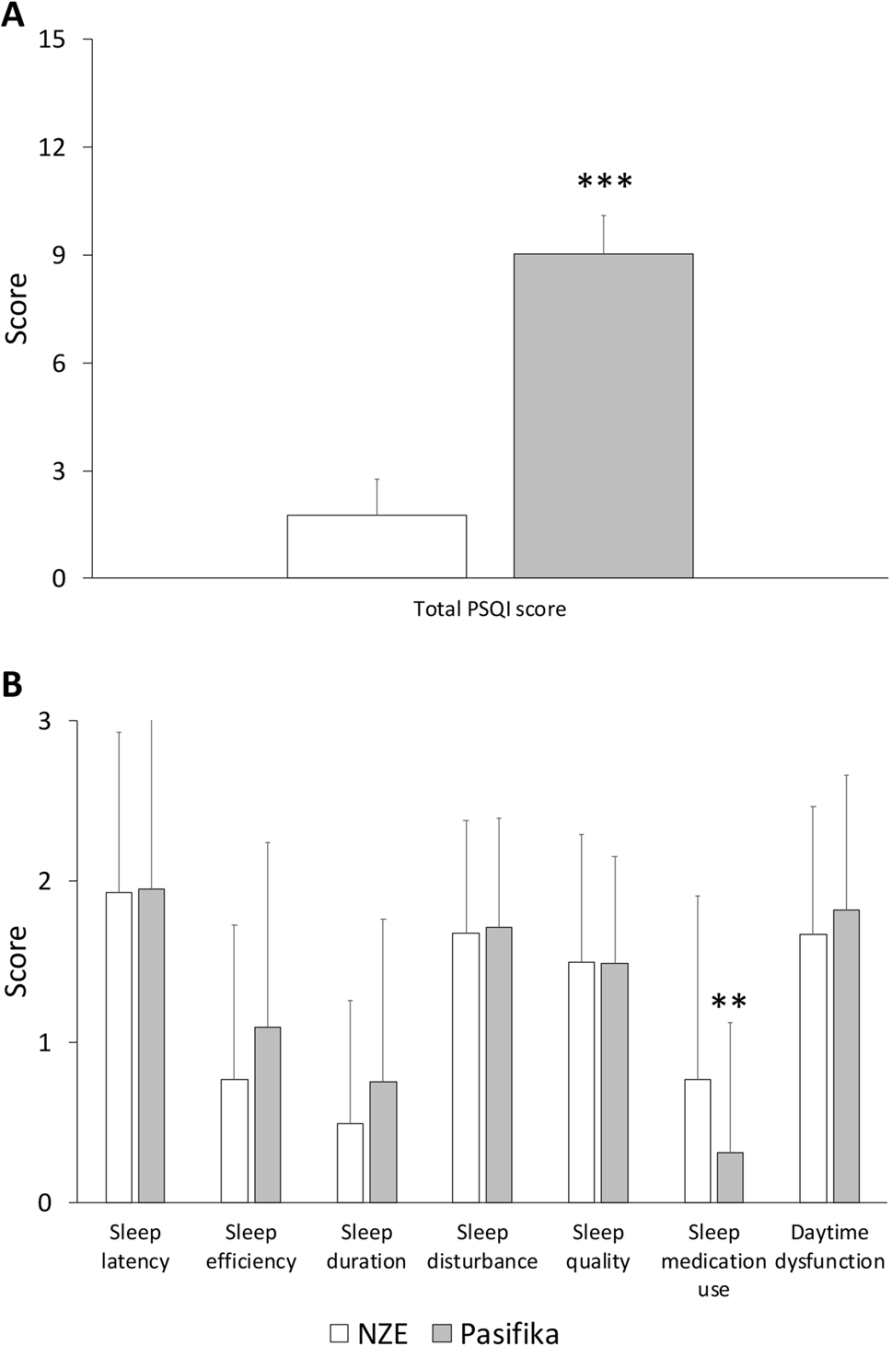
The comparison of PSQI (**A**) and its components (**B**) scores between NZE (white bars) and Pasifika (grey bars) students. Pasifika students rated higher overall PSQI (**A**) but lower medication use (**B**) scores than NZE students. Other PSQI components were similar between ethnicities. Error bars indicate standard deviations. **Significantly different from NZE students, *P* < .01, ***P < .001

The perceived academic stress and its subscales were comparable between Pasifika and NZE students (Fig. 1). Pasifika students, however, had poorer overall sleep quality than NZE students (Fig. 2A, t(113) = 14.41, *P *< .001). In terms of the sleep quality subscales, most were similar between ethnicities, except for the use of sleep medication which was more frequent among NZE students than Pasifika students (Fig. 2B, t(113) = 2.457, *P* = .008).

**Fig. 3:**
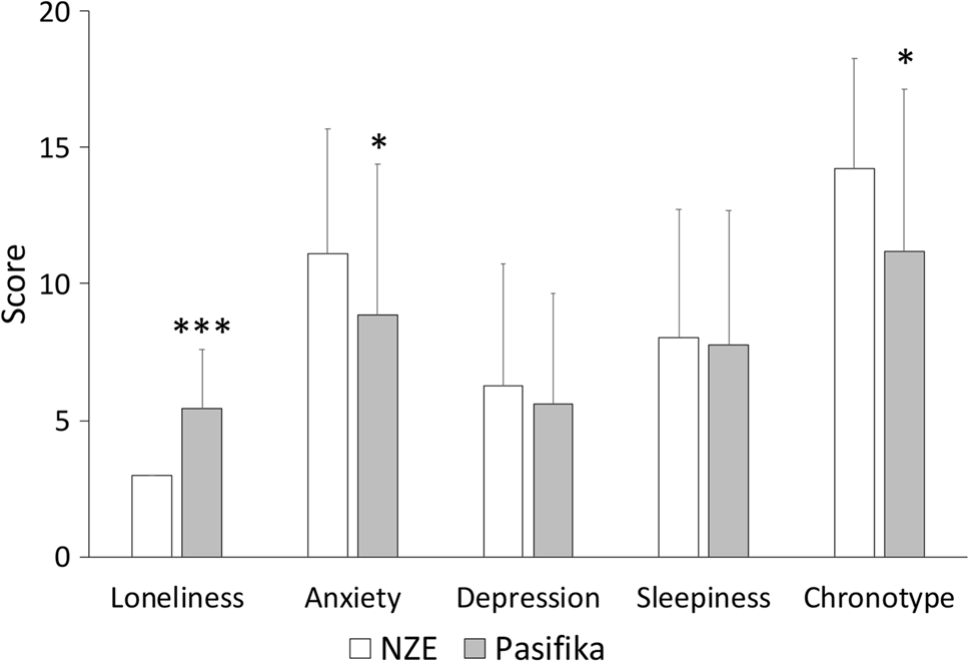
Self-reported levels of loneliness, anxiety, depression, daytime sleepiness and chronotype of NZE (grey bars) and Pasifika (white bars) students. Their scores for depressive symptoms and daytime sleepiness were comparable. However, Pasifika students reported more loneliness, less anxiety symptoms, and less of a morning person than NZE students. Error bars indicate standard deviations. *Significantly different from NZE students, *P* < .05; ***P < .001

As shown on Fig. 3, depressive symptoms and daytime sleepiness were similar among students, regardless of their ethnicities. In contrast, Pasifika students reported more loneliness (t(119) = 8.933, *P *< .001) and less anxiety symptoms (t(120) = 2.469, *P* = .015). Pasifika students also felt less of a morning person (t(121) = 2.618, *P* = .010) than NZE students, but they had similar bedtime, wake time, time in bed, and sleep amount (Table 2).

**Table 2:** Average bed time, waking time, time in bed, and sleep amount of Pasifika and NZE students. Data are shown as means (standard deviation)

	NZE	Pasifika	Test statistics	P values
Bed time	23:31 (0:25)	00:08 (0:07)	t(117) = 1.906	.059
Wake time	8:01 (0:21)	8:24 (0:06)	t(117) = 1.165	.246
Time in Bed (hours)	8.51 (1.29)	8.26 (1.83)	t(117) = .871	.385
Sleep amount (hours)	7.07 (1.21)	6.70 (1.26)	t(115) = 1.649	.102

### Correlation Analyses

Table 3 shows that academic stress correlated with poorer sleep quality (*r* = .279, *P* = .003), loneliness (*r* = .304, *P *< .001), anxiety symptoms (*r* = .526, *P *< .001), and depressive symptoms (*r* = .551, *P *< .001). In addition, poor sleep quality correlated with loneliness (*r* = .780, *P *< .001), depressive symptoms (*r* = .252, *P* = .007), and eveningness (*r* = -.319, *P* < .001).

**Table 3:** Correlation matrix between academic stress, sleep and psychological wellbeing

	Perceived academic stress	PSQI score	Loneliness	Anxiety	Depression	Sleepiness	Chronotype
Perceived academic stress	1	.279**	.304***	.526***	.551***	.075	-.088
PSQI score		1	.780**	.135	.252**	.129	-.190*
Loneliness			1	.233*	.273**	.071	.023
Anxiety				1	.736**	.485**	.319**
Depression					1	.430**	.103
Sleepiness						1	.310*
Chronotype							1

*Significant correlation

*P* < .05

**P < .01

***P < .001

### Mediation Analyses

**Fig. 4:**
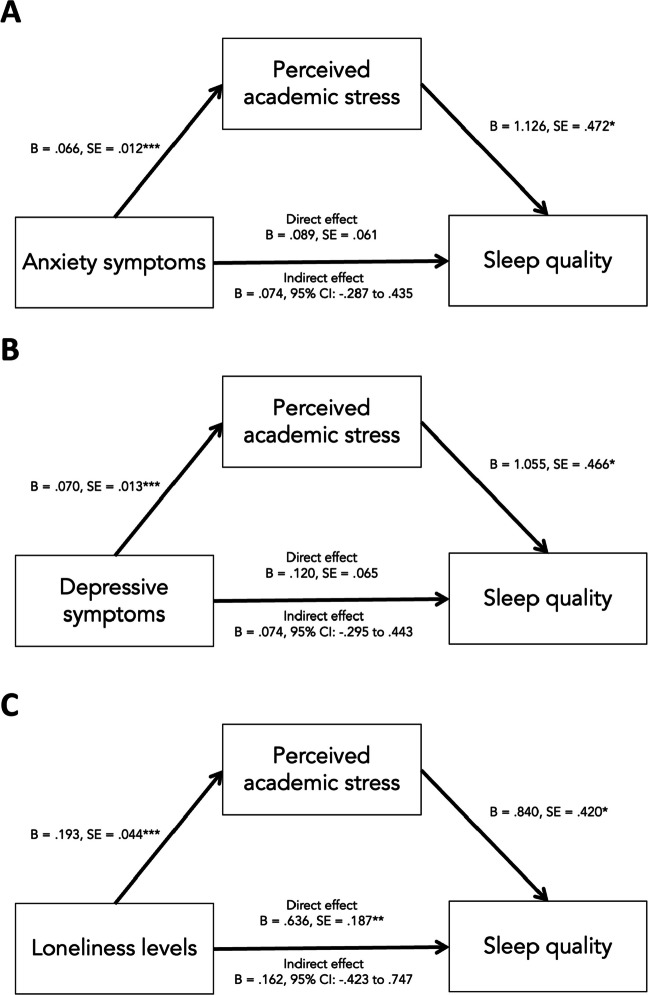
Mediation models testing the effects of anxiety symptoms (**A**), depressive symptoms (**B**), and loneliness (**C**) on sleep quality through their influence on academic stress. The direct effects of anxiety and depressive symptoms on sleep quality were no longer significant but the direct effect of loneliness on sleep quality remained significant, when academic stress was added as a mediator. *P < .05; **P < .01, CI: Confidence interval, SE: Standard error

Figure 4 shows how academic stress mediated the relationships between psychological wellbeing and sleep quality, after controlling for age, ethnicity and gender. The total effect of anxiety symptoms on sleep quality was .066 (95% CI: 0.42 to .089, *P* < .001). The direct effect of anxiety symptoms on sleep quality was .089 (95% CI: -.032 to .210), after adjusting for academic stress. The indirect effect of anxiety symptoms on sleep quality through academic stress was .074 (95% CI: -.287 to .435, *P* < .05).

The total effect of depressive symptoms on sleep quality was .195 (95% CI: 0.81 to .309, *P* < .01). The direct effect of depressive symptoms on sleep quality was .120 (95% CI: -.009 to .250), after adjusting for academic stress. The indirect effect of depressive symptoms on sleep quality through academic stress was .074 (95% CI: -.295 to .443, * P* < .05).

Loneliness partially mediated the relationship between loneliness and sleep quality. The total effect of loneliness on sleep quality was .802 (95% CI: 0.463 to 1.141, *P* < .001). The direct effect of loneliness on sleep quality was .636 (95% CI: .264 to 1.008), after adjusting for academic stress.

## Discussion

In this study, we found that academic stress levels were similar among NZE and Pasifika students. However, Pasifika students reported worse sleep quality, less severe anxiety symptoms, lonelier, and less of a morning person than NZE students. Additionally, academic stress was found to mediate the relationship between psychological wellbeing and sleep quality among students. Our finding emphasizes that academic stress is associated with students’ wellbeing, and that institutions need to have a support infrastructure for students’ wellbeing. Such a support needs to also be inclusive and welcoming to underrepresented ethnic minority students.

### Academic Stress

We are surprised to find that both Pasifika and NZE students reported similar level of academic stress. As previously reported, Pasifika students in our department on average achieved lower academic marks in anatomy courses than NZE students [14]. Thus, we expected that Pasifika students might have higher academic stress than NZE students. However, our finding is similar to what is reported by Barbayannis et al. [39], in which no ethnic differences is found in academic stress among university students in the Unites States. One potential explanation is that ethnic minority students may have stigma in reporting mental health issues. As an example, one study in the United States indicates that the rate of mental health diagnosis is lower among ethnic minority students than White students, but their rate of suicide ideation or attempts may be higher [40].

It is also worth noting that other studies have indicated elevated academic stress among ethnic minority students. For example, being Latino in a pre-dominantly White university is associated with higher academic stress, including stresses related to perform well, workload and self-perceptions [41]. Similarly, African American students experience more profound stress than White students in a predominantly-White institution [42].

Demographic differences may potentially explain the inconsistencies in the findings. The study by Barbayannis et al. [39] involved an online recruitment of students in the United States through Prolific and is not restricted to a specific institution, which may potentially confound their data on students’ stress. In contrast, the two studies mentioned in the last paragraph [41, 42] were done in White-majority universities, so the students were studying as ethnic minority students. Previously, we have noted that social anxiety as ethnic minority correlates with academic stress among Pasifika students in New Zealand [19]. Thus, studies on ethnic difference in academic stress need to take into account the setting of the institutions where they study (e.g., whether students are studying as an ethnic minority or not).

### Sleep Quality

In this study, the overall self-reported sleep quality was poorer in Pasifika students than in NZE students. This finding supports our previous study where over 70% of Pasifika students in our department report having insomnia symptoms [21]. In that study, 31.6% report moderate and 7% report severe insomnia symptoms. Furthermore, that study also indicates that poor sleep hygiene mediates the relationship between academic stress and insomnia symptoms among Pasifika students. Some of the poor sleep hygiene that correlate with insomnia symptoms are inconsistent sleep/wake time, staying in bed longer than necessary, being distressed or worried while in bed [21]. These results suggest the importance of having sleep education for Pasifika students in order to improve their sleep health. A workshop or information session could potentially be offered to students on how to manage their sleep and wellbeing during their studies.

In analyzing the components of PSQI, we found that NZE students took sleep medication more often than Pasifika students. This finding may help explain that NZE reported better sleep because of their sleep medication use. The difference in sleep medication use also suggests that awareness and attitudes towards sleep medication vary between NZE and Pasifika students. NZE students may be more exposed to and accepting of pharmacological solutions for sleep issues, because there is evidence from the general population that Pacific peoples may be less likely to access medicines than NZE [43]. Cost may be a contributor to this difference, as Pasifika students are more likely to have come from a lower income household than NZE students [44].

### Psychological Wellbeing

Here we showed that Pasifika students reported more loneliness than NZE students. Most Pasifika students in our institution come from outside Dunedin [14, 19, 21], majority from regions in New Zealand that have much higher proportions of Pacific communities. Some of them also report of experiencing a culture shock when they move to Dunedin because of the proportionally fewer Pacific people they meet in Dunedin [15]. In moving to Dunedin, they need to form new connections. However, Dunedin has less than 3% Pacific population [45] so making new connections with other Pacific peoples (commonly through Pacific student associations or church involvement) may be challenging for some Pasifika students. In addition, some Pasifika students in Dunedin may feel lonely because they are away from their families, especially that family is an important aspect of daily living among Pacific peoples. Furthermore, there are few Pasifika academics and staff in the University of Otago [46] so Pasifika students may not receive culturally-appropriate support that they need. These factors may lead them to feel lonely while studying.

Our finding on depressive and anxiety symptoms is in contrast to a finding from a past study where ethnic minority students in a predominantly-White university in the United States report higher odds of having depressive symptoms, but similar anxiety symptoms, than White students [47]. In that study, however, the authors group all participants who are not White as ethnic minority students in their analyses, and they also use different depression and anxiety scales than the ones we use.

It remains unclear why Pasifika students reported less anxiety symptoms than NZE students. It is worth noting that the anxiety scale we use is for assessing general anxiety. The finding may potentially be different if we were to assess social anxiety or anxiety related to academic works. Admittedly, Pasifika students are present as ethnic minority students in our institution, and there are few Pasifika academics in our institution [46]. In our previous study [19], we show that Pasifika students in our department may experience social anxiety as ethnic minority, and that their social anxiety correlates with their academic stress. Unfortunately, Pacific peoples, both students and staff, may experience discrimination in academic setting [46, 48]. Such an experience may potentially lead to more social anxiety as an ethnic minority.

Our results on students’ chronotype is worth further discussion. While Pasifika students felt they were more of an evening person than NZE students, their self-reported sleep/wake times were comparable. It would be interesting for a future study to confirm their sleep/wake times using objective measures like actigraphy or polysomnography. However, our finding reflects data from another study where Pasifika adolescents report having a later bedtime than NZE adolescents [25]. We did not find an ethnic difference in bedtime in our study, potentially because our cohort is older than the ones in the Galland et al. study [25]. The study by Galland et al. [25] also reports that Pasifika adolescents are more likely to do school works, do household chores, have caffeinated drinks, and listen to music close to bedtime than NZE adolescents. It remains to be investigated if these pre-sleep habits persist when they study as university students. One factor which will be different is that they are likely to live without their family so their pre-sleep habits may change once they are university students.

### Supporting Student Wellbeing

Our data stress the importance for academic institutions to have an infrastructure to support student wellbeing during their studies. Resources like mental health support are critical for students’ wellbeing. Furthermore, following the COVID-19 pandemic, students’ mental health may be impacted, but universities may not be equipped to meet the increased demand (e.g., due to lack of staff or funding). We also acknowledge that cultural background and other factors may influence students’ willingness to access mental health support. Ethnic minority individuals, for example, prefer to approach therapists of the same ethnicity for psychological counselling [49]. Thus, it is important for academic institutions to have staff who are culturally competent when meeting students of ethnic minority [50].

### Limitation

Our study has several limitations. For example, we did not include an objective measure for sleep assessment like polysomnography or actigraphy. However, all questionnaire measures have been validated, including the PSQI which have been validated in a university student population [51]. In addition, as Pasifika students are ethnic minority students, their numbers in our department are relatively fewer than NZE students, and thus we are unable to perform sub-analyses on the wellbeing or academic stress of specific Pacific ethnicities. Furthermore, we recruited Pasifika students with multiple ethnicities as well, which may potentially influence the results as their experiences may not always be the same as Pasifika students with a single ethnicity. While not assessed in this study, future studies could include a question on the level of strength of ethnic identity. We also acknowledge that we use a convenience sampling strategy by recruiting students in our department. There may be sampling bias as many of the students are familiar with our team members. However, other than the email invitation, we did not approach the participants in-person. Our recruitment strategy may also limit the generalizability of the finding, i.e., the findings may not be replicable for students in other departments or universities. We also recognize that sleep and psychological wellbeing are complex, and factors other than academic stress that we did not assess may influence both of them.

### Conclusion

Pasifika and NZE anatomy students in New Zealand do not always have the same experience during their study in higher education. While their academic stress levels are similar, the psychological wellbeing of Pasifika students are more affected during their studies. Institutions in New Zealand should provide support to students, not just academically but also for their wellbeing.

## Data Availability

Data are not deposited in a repository.

## References

[1] Bedewy D, Gabriel A. Examining perceptions of academic stress and its sources among university students: the Perception of Academic Stress Scale. Health Psychol Open. 2015;2:2055102915596714. 28070363 10.1177/2055102915596714 PMC5193280

[2] Duthie CJ, Cameron C, Smith-Han K, Beckert L, Delpachitra S, Garland SN, et al. Sleep management strategies among medical students at the University of Otago. Behav Sleep Med. 2022;21:448–59. 36178287 10.1080/15402002.2022.2127723

[3] Samaranayake CB, Arroll B, Fernando AT 3rd. Sleep disorders, depression, anxiety and satisfaction with life among young adults: a survey of university students in Auckland, New Zealand. N Z Med J. 2014;127:13–22. 25145302

[4] Azad MC, Fraser K, Rumana N, Abdullah AF, Shahana N, Hanly PJ, Turin TC. Sleep disturbances among medical students: a global perspective. J Clin Sleep Med. 2015;11:69–74. 25515274 10.5664/jcsm.4370 PMC4265662

[5] Duthie CJ, Cameron C, Smith-Han K, Beckert L, Delpachitra S, Garland SN, et al. Reasons for why medical students prefer specific sleep management strategies. Behav Sleep Med. 2024. 10.1080/15402002.2024.2318261. 10.1080/15402002.2024.2318261 38369858

[6] Jones RD. Ethnoracial sleep disparities among college students living in dormitories in the United States: a nationally representative study. Sleep Health. 2020;6:40–7. 31759933 10.1016/j.sleh.2019.10.005 PMC6995403

[7] Lukowski AF, Kamliot DZ, Schlaupitz CA. Insomnia and behaviorally induced sleep syndrome in undergraduates tested during the COVID-19 pandemic: associations with health, stress, and GPA. J Clin Sleep Med. 2024;20:261–9. 37858288 10.5664/jcsm.10844 PMC10835785

[8] Sa J, Samuel T, Chaput JP, Chung J, Grigsby-Toussaint DS, Lee J. Sex and racial/ethnic differences in sleep quality and its relationship with body weight status among US college students. J Am Coll Health. 2020;68:704–11. 31039082 10.1080/07448481.2019.1594829

[9] Mong JA, Cusmano DM. Sex differences in sleep: impact of biological sex and sex steroids. Philos Trans R Soc Lond B Biol Sci. 2016;371:20150110. 26833831 10.1098/rstb.2015.0110 PMC4785896

[10] Sa J, Choe S, Cho BY, Chaput JP, Kim G, Park CH, et al. Relationship between sleep and obesity among U.S. and South Korean college students. BMC Public Health. 2020;20:96. 31969131 10.1186/s12889-020-8182-2 PMC6977299

[11] Tsui YY, Wing YK. A study on the sleep patterns and problems of university business students in Hong Kong. J Am Coll Health. 2009;58:167–76. 19892654 10.1080/07448480903221418

[12] Stats NZ. Pacific Peoples ethnic group. 2018. https://www.stats.govt.nz/tools/2018-census-ethnic-group-summaries/pacific-peoples. Accessed on 23 May 2024.

[13] Anae AM, Mila-Schaaf K, Coxon E, Mara D, Sanga K. Teu Le Va - Relationships across research and policy in Pasifika education. Ministry of Education. 2010. https://www.educationcounts.govt.nz/__data/assets/pdf_file/0009/75897/944_TeuLeVa-30062010.pdf

[14] Time WS, Samalia L, Wibowo E. Anatomical sciences education needs to promote academic excellence of ethnic minority students-evidence from Pasifika students at the University of Otago. Anat Sci Educ. 2023;16:1011–23. 37501349 10.1002/ase.2319

[15] Fakapulia IF. An in-depth look into the education experiences of Pasifika anatomy students: University of Otago; 2022.

[16] Wikaire E, Curtis E, Cormack D, Jiang Y, McMillan L, Loto R, Reid P. Predictors of academic success for Māori, Pacific and non-Māori non-Pacific students in health professional education: a quantitative analysis. Adv Health Sci Educ. 2017;22:299–326. 10.1007/s10459-017-9763-4 28236125

[17] Sopoaga F, Zaharic T, Kokaua J, Ekeroma AJ, Murray G, van der Meer J. Pacific students undertaking the first year of health sciences at the University of Otago, and factors associated with academic performance. N Z Med J. 2013;126:96–108. 24162634

[18] Brown SJ, Power N, Bowmar A, Foster S. Student engagement in a Human Anatomy and Physiology course: a New Zealand perspective. Adv Physiol Educ. 2018;42:636–43. 30303414 10.1152/advan.00035.2018

[19] Fakapulia IF, Samalia L, Wibowo E. Social factors are associated with academic stress in Pasifika students at the University of Otago. Waikato J Educ. 2023;28:125–39.

[20] Fakapulia IF, Time WS, TuiSamoa G, Samalia L, Wibowo E. Does religiosity play a role in anatomy learning?. Perspectives from Pasifika students at the University of Otago. Anatomical Sciences Education; 2024. 10.1002/ase.2380 38291614

[21] Fakapulia IF, Samalia L, Wibowo E. Sleep hygiene mediates the relationship between perceived academic stress and insomnia symptom severity among Pasifika students in Aotearoa New Zealand NZ J Psychol. 2024;52:23–9.

[22] Lee CHJ, Sibley CG. Sleep duration and psychological well-being among New Zealanders. Sleep Health. 2019;5:606–14. 31377250 10.1016/j.sleh.2019.06.008

[23] Fernando AT, Samaranayake CB, Blank CJ, Roberts G, Arroll B. Sleep disorders among high school students in New Zealand. J Prim Health Care. 2013;5:276–82. 24294615

[24] McLay L, Tautolo E, Iusitini L, Richards R, Galland B, Schluter PJ. The relationship between sleep duration and health among Pacific adolescents within New Zealand: findings from the Pacific Islands families study. Aust NZ J Public Health. 2023;47:100021. 10.1016/j.anzjph.2023.100021 36917880

[25] Galland BC, de Wilde T, Taylor RW, Smith C. Sleep and pre-bedtime activities in New Zealand adolescents: differences by ethnicity. Sleep Health. 2020;6:23–31. 31699636 10.1016/j.sleh.2019.09.002

[26] Dorofaeff TF, Denny S. Sleep and adolescence. Do New Zealand teenagers get enough? J Paediatr Child Health. 2006;42:515–20. 16925537 10.1111/j.1440-1754.2006.00914.x

[27] Dinis J, Braganca M. Quality of sleep and depression in college students: a systematic review. Sleep Sci. 2018;11:290–301. 30746048 10.5935/1984-0063.20180045 PMC6361309

[28] Nyer M, Farabaugh A, Fehling K, Soskin D, Holt D, Papakostas GI, et al. Relationship between sleep disturbance and depression, anxiety, and functioning in college students. Depress Anxiety. 2013;30:873–80. 23681944 10.1002/da.22064 PMC3791314

[29] Ulrich AK, Full KM, Cheng B, Gravagna K, Nederhoff D, Basta NE. Stress, anxiety, and sleep among college and university students during the COVID-19 pandemic. J Am Coll Health. 2023;71:1323–7. 34242544 10.1080/07448481.2021.1928143 PMC8742838

[30] Griffin SC, Williams AB, Ravyts SG, Mladen SN, Rybarczyk BD. Loneliness and sleep: a systematic review and meta-analysis. Health Psychol Open. 2020;7:2055102920913235. 32284871 10.1177/2055102920913235 PMC7139193

[31] Hom MA, Chu C, Rogers ML, Joiner TE. A meta-analysis of the relationship between sleep problems and loneliness. Clin Psychol Sci. 2020;8:799–824.

[32] Bradford DRR, Biello SM, Russell K. Insomnia symptoms mediate the association between eveningness and suicidal ideation, defeat, entrapment, and psychological distress in students. Chronobiol Int. 2021;38:1397–408. 34100311 10.1080/07420528.2021.1931274

[33] Buysse DJ, Reynolds CF 3rd, Monk TH, Berman SR, Kupfer DJ. The Pittsburgh Sleep Quality Index: a new instrument for psychiatric practice and research. Psychiatry Res. 1989;28:193–213. 2748771 10.1016/0165-1781(89)90047-4

[34] Hughes ME, Waite LJ, Hawkley LC, Cacioppo JT. A short scale for measuring loneliness in large surveys: results from two population-based studies. Res Aging. 2004;26:655–72. 18504506 10.1177/0164027504268574 PMC2394670

[35] Zigmond AS, Snaith RP. The hospital anxiety and depression scale. Acta Psychiatr Scand. 1983;67:361–70. 6880820 10.1111/j.1600-0447.1983.tb09716.x

[36] Adan A, Almirall H. Horne & Ostberg morningness-eveningness questionnaire: a reduced scale. Pers Individ Dif. 1991;12:241–53.

[37] Johns MW. A new method for measuring daytime sleepiness: the Epworth sleepiness scale. Sleep. 1991;14:540–5. 1798888 10.1093/sleep/14.6.540

[38] Baron RM, Kenny DA. The moderator-mediator variable distinction in social psychological research: conceptual, strategic, and statistical considerations. J Pers Soc Psychol. 1986;51:1173–82. 3806354 10.1037//0022-3514.51.6.1173

[39] Barbayannis G, Bandari M, Zheng X, Baquerizo H, Pecor KW, Ming X. Academic stress and mental well-being in college students: correlations, affected groups, and COVID-19. Front Psychol. 2022;13:886344. 35677139 10.3389/fpsyg.2022.886344 PMC9169886

[40] Liu CH, Stevens C, Wong SHM, Yasui M, Chen JA. The prevalence and predictors of mental health diagnoses and suicide among U.S. college students: implications for addressing disparities in service use. Depress Anxiety. 2019;36:8–17. 30188598 10.1002/da.22830 PMC6628691

[41] Huynh HP, Sifuentes KA, Lilley MK. Context matters: stress for minority students who attend minority-majority universities. Psychol Rep. 2023;126:246–64. 34617864 10.1177/00332941211043459

[42] Negga F, Applewhite S, Livingston I. African American college students and stress: school racial composition, self-esteem and social support. Coll Stud J. 2007;41:823–30.

[43] Metcalfe S, Laking G, Arnold J. Variation in the use of medicines by ethnicity during 2006/07 in New Zealand: a preliminary analysis. N Z Med J. 2013;126:14–41. 24162628

[44] Stats NZ. Household income and housing-cost statistics: Year ended June 2023. 2024. https://www.stats.govt.nz/information-releases/household-income-and-housing-cost-statistics-year-ended-june-2023/. Accessed on 23 May 2024.

[45] Stats NZ. Dunedin City. 2018. https://www.stats.govt.nz/tools/2018-census-place-summaries/dunedin-city#ethnicity-culture-and-identity. Accessed on 23 May 2024.

[46] Naepi S. Why isn’t my professor Pasifika? A snapshot of the academic workforce in New Zealand universities. MAI J. 2019;8:220–34.

[47] Rajbhandari-Thapa J, Chiang K, Lee MC, Treankler A, Padilla H, Vall EA, Fedrick M. Depression and anxiety among college students at Historically Black and Predominantly White universities during the COVID-19 pandemic: a cross-sectional study. J Am Coll Health. 2023. 10.1080/07448481.2023.2230297. 10.1080/07448481.2023.2230297 37487205

[48] McAllister T, Naepi S, Walker L, Gillon A, Clark P, Lambert E, et al. Seen but unheard: navigating turbulent waters as Māori and Pacific postgraduate students in STEM. J R Soc N Z. 2022;52:116–34.

[49] Huang CY, Zane N. Cultural influences in mental health treatment. Curr Opin Psychol. 2016;8:131–6. 29506788 10.1016/j.copsyc.2015.10.009 PMC9528809

[50] Sanchez K, Ybarra R, Chapa T, Martinez ON. Eliminating behavioral health disparities and improving outcomes for racial and ethnic minority populations. Psychiatr Serv. 2016;67:13–5. 26325461 10.1176/appi.ps.201400581

[51] Dietch JR, Taylor DJ, Sethi K, Kelly K, Bramoweth AD, Roane BM. Psychometric evaluation of the PSQI in US college students. J Clin Sleep Med. 2016;12:1121–9. 27166299 10.5664/jcsm.6050 PMC4957190

